# Complex and dynamic patterns of Wnt pathway gene expression in the developing chick forebrain

**DOI:** 10.1186/1749-8104-4-35

**Published:** 2009-09-04

**Authors:** Robyn Quinlan, Manuela Graf, Ivor Mason, Andrew Lumsden, Clemens Kiecker

**Affiliations:** 1 MRC Centre for Developmental Neurobiology, New Hunt's House, Guy's Hospital Campus, King's College, London SE1 1UL, UK

## Abstract

**Background:**

Wnt signalling regulates multiple aspects of brain development in vertebrate embryos. A large number of *Wnt*s are expressed in the embryonic forebrain; however, it is poorly understood which specific Wnt performs which function and how they interact. Wnts are able to activate different intracellular pathways, but which of these pathways become activated in different brain subdivisions also remains enigmatic.

**Results:**

We have compiled the first comprehensive spatiotemporal atlas of Wnt pathway gene expression at critical stages of forebrain regionalisation in the chick embryo and found that most of these genes are expressed in strikingly dynamic and complex patterns. Several expression domains do not respect proposed compartment boundaries in the developing forebrain, suggesting that areal identities are more dynamic than previously thought. Using an *in ovo *electroporation approach, we show that *Wnt4 *expression in the thalamus is negatively regulated by Sonic hedgehog (Shh) signalling from the zona limitans intrathalamica (ZLI), a known organising centre of forebrain development.

**Conclusion:**

The forebrain is exposed to a multitude of Wnts and Wnt inhibitors that are expressed in a highly dynamic and complex fashion, precluding simple correlative conclusions about their respective functions or signalling mechanisms. In various biological systems, Wnts are antagonised by Shh signalling. By demonstrating that *Wnt4 *expression in the thalamus is repressed by Shh from the ZLI we reveal an additional level of interaction between these two pathways and provide an example for the cross-regulation between patterning centres during forebrain regionalisation.

## Background

The vertebrate forebrain is the most functionally complex of biological structures and the centre of all higher brain functions, including learning, memory, cognition and, in humans, self-awareness, reasoning and personality. During embryogenesis, the forebrain is induced at gastrula stages when gradients of signalling molecules establish anteroposterior (AP) and dorsoventral (DV) polarity in the neural plate [1-5]. One such signal is mediated by members of the Wnt family of secreted glycoproteins that induce posterior and suppress anterior neural identity in a dose-dependent manner [6-8]. The prospective forebrain is protected from posteriorising Wnt signals by various Wnt pathway antagonists that are expressed in the anterior neuroectoderm and in the underlying anterior axial mesendoderm [9-17].

Following neural induction, the prospective forebrain becomes progressively regionalised. The first major division is the establishment of the telencephalon anteriorly and of the diencephalon posteriorly. The dorsal telencephalon gives rise to the pallium (including the hippocampus and the cerebral cortex in mammals) while the ventral part (subpallium) gives rise to the striatum and globus pallidus (lateral and medial ganglionic eminences). The hypothalamus evolves from neuroepithelium that is located ventral to the subpallium. The diencephalon consists of three major AP subdivisions: prethalamus, thalamus and pretectum (from anterior to posterior). Based on their topographical relationship within the postnatal brain, prethalamus and thalamus were previously referred to as ventral thalamus and dorsal thalamus, respectively. However, these terms obscure their embryonic origins as AP subdivisions of the diencephalic primordium. The area dorsal to the prethalamus constitutes the eminentia thalami; dorsal to the thalamus lies the epithalamus (habenula) and the dorsal part of the pretectum forms the posterior commissure. The epiphysis (pineal gland), a small endocrine gland that secretes melatonin and modulates wake/sleep patterns, is located at the dorsal midline of the epithalamus. For a detailed topographical description of forebrain subdivisions see [18].

We are only beginning to understand the mechanisms that generate the complex regional diversity of the forebrain. As in other parts of the vertebrate neural tube, groups of forebrain cells are set aside to function as local signalling centres ('organisers') that regulate patterning and proliferation in adjacent areas [19,20]. Telencephalic regionalisation is regulated by three such signalling centres: the commissural plate at the anterior pole of the forebrain that secretes fibroblast growth factors (Fgfs), the ventralmost aspect of the telencephalon (lamina terminalis) that secretes the morphogen Sonic hedgehog (Shh) and the dorsal border of the pallium ('cortical hem' in mammals) that releases bone morphogenetic proteins (Bmps) and Wnts [21-23]. Furthermore, members of several signalling molecule families are expressed along the boundary between the pallium and the subpallium (PSB) [24].

In the diencephalon, *Shh *is expressed throughout the basal plate and at later stages also in the the zona limitans intrathalamica (ZLI), a stripe of cells that interfaces the prethalamus and the thalamus and acts as an organiser of thalamic development [25-29]. Cell lineage labelling experiments in chick embryos have suggested that the ZLI is derived from a wedge-shaped, *Lunatic fringe *(*Lfng*)-negative area in the early diencephalon [30]. The diencephalic primordium as a whole expands significantly before and during ZLI formation whereas the *Lfng*-negative wedge that gives rise to the ZLI seemingly narrows along its AP axis and elongates along its DV axis. *Shh *expression is subsequently seen within this territory as a characteristic sharp peak between the prethalamus and thalamus [30]. Neither the mechanism nor the functional significance of this striking allometric growth are known.

Wnt signalling has been implicated in multiple aspects of central nervous system development, ranging from early pattern formation to the establishment of axonal connectivity [31]. After its initial role in antagonising anterior development [6-17,32], Wnt signalling is required for the establishment of the posterior forebrain [33-35], including the thalamus [36]. Various Wnts are expressed along the dorsal border of the pallium/cortical hem while *Sfrp2*, encoding the putative Wnt inhibitor secreted Frizzled-related protein (Sfrp) 2, is expressed along the PSB, raising the possibility that a gradient of Wnt signalling is established across the cortex/pallium between the cortical hem and the PSB (hence the alternative name 'anti-hem' for the PSB) [24]. In the chick telencephalon, *Sfrp1 *is expressed in a similar fashion at late developmental stages [37]. Wnt signalling, in conjunction with Fgfs, specifies dorsal telencephalic fate early on [38,39] and hippocampal fate at later stages [40-43]. However, there is no evidence for a direct role of Wnt signalling in the arealisation of the cortex; instead, the main function of Wnts in this tissue appears to be the regulation of neurogenesis [43-48].

Although Wnt signalling has been shown to influence multiple aspects of brain development, few studies have succeeded in linking a specific developmental process to a single Wnt ligand, possibly due to extensive functional redundancy within the Wnt family. In this study, we have analysed the expression of a large number of Wnt ligands, Wnt receptors and intracellular targets of the Wnt signalling pathway during critical stages of forebrain regionalisation. We find that no fewer than twelve Wnts, seven Wnt receptors, three extracellular and three intracellular Wnt inhibitors as well as five nuclear factors that regulate the transcriptional output of the Wnt pathway are expressed in characteristic, regionalised and dynamic patterns in the developing chick forebrain. For selected genes, we performed double *in situ *hybridisation to relate their expression to known developmental domains within the diencephalon and telencephalon. Interestingly, we found that *Wnt4 *is strongly expressed in the emerging thalamus, but becomes downregulated with the appearance of *Shh *expression in the ZLI. To further probe the regulatory relationship suggested by this expression, we performed *in ovo *eletroporation experiments to demonstrate that Shh signalling is both necessary and sufficient for the downregulation of *Wnt4*, indicating that the organising function of the ZLI may be mediated not only directly, through Shh signalling, but also indirectly, by regulating other diffusible signals. Our study highlights the potential complexity of Wnt signalling during forebrain development, lays the foundation for a systematic functional analysis of Wnt signalling in this area and reveals a novel regulatory interaction between the Wnt and Shh signalling pathways.

## Materials and methods

### Chick embryos

Fertilised hens' eggs (Henry Stewart Ltd, Louth, Lincolnshire, UK) were incubated in a humidified chamber at 38°C until they reached the desired stages. Embryos were staged according to the tables by Hamburger and Hamilton (HH) [49] and fixed over night at 4°C in 4% paraformaldehyde in phosphate buffered saline.

### *In situ *hybridisation

Gene expression analysis by *in situ *hybridisation was performed as described elsewhere [50]. The references for the *Wnt in situ *constructs can be found in [51]. The following clones were obtained from the chick expressed sequence tag database, linearised with *Not*I and transcribed with T3 RNA polymerase to generate probes for *in situ *hybridisation: *Axin1*, ChEST175L2; *Axin2*, ChEST755b16; *Axud1*, ChEST782g10; *Ctbp1*, ChEST765f10; *Ctbp2*, ChEST411c2; *Drapc1*, ChEST357h24; *Idax*, ChEST644a15.

### *In ovo *electroporation and immunochemical detection of green fluorescent protein expression

HH13 to HH15 embryos were electroporated *in ovo *as described [25] with expression plasmids driving the expression of Shh (pXEX-Shh [52]) or Ptc^Δloop2 ^[53]. The Shh-expressing construct was co-electroporated with a green fluorescent protein (GFP)-expressing plasmid to allow for retrospective localisation of the electroporated area. The eggs were resealed using adhesive tape and incubated at 38.5°C for 1 to 2 days. Embryos were dissected, fixed as described above and subjected to *in situ *hybridisation. After *in situ *hybridisation, GFP expression was detected using a polyclonal antibody against GFP and a fluorescently labelled (Alexa) secondary anti-rabbit IgG antibody (both Molecular Probes, Invitrogen Ltd, Paisley, UK) using standard methods [25].

## Results

In order to investigate the role of Wnt signalling during vertebrate forebrain regionalisation, we initially set out to generate a spatiotemporal atlas of Wnt pathway gene expression in the embryonic chick forebrain. A comprehensive analysis of *Wnt *expression during early neural plate stages has been previously performed [51]. Thus, we focussed on developmental stages after neural tube closure when characteristic morphological constrictions and bulges emerge within the forebrain neuroepithelium (HH13 to HH24) [20]. These stages are crucial for the regionalisation of the diencephalons, including the formation of the ZLI [30,54] and for the establishment of subpallial and hypothalamic subdivisions [55,56]. Additionally, the entire amniote forebrain undergoes substantial growth around this time, but various regions of the forebrain proliferate differentially, suggesting a dynamic regulation of growth-promoting cues.

We have loosely organised our *Wnt *expression data in three groups: those *Wnts *whose expression is restricted to the dorsalmost forebrain (*Wnt1 *and *Wnt6*), those that are expressed more widely in the dorsal half of the forebrain, but are not detected ventrally (*Wnt3*, *Wnt3A *and *Wnt4*), and those whose expression also extends into the ventral forebrain (*Wnt2B/13*, *Wnt5A*, *Wnt5B*, *Wnt7A*, *Wnt7B*, *Wnt8B *and *Wnt9A/14*; see [57] for nomenclature of vertebrate *Wnt *genes). We did not observe any forebrain expression of *Wnt2 *[58], *Wnt8 *[59], *Wnt11 *[60] or *Wnt11B *[61] at the stages examined.

### *Wnt1 *and *Wnt6*

**Figure 1:**
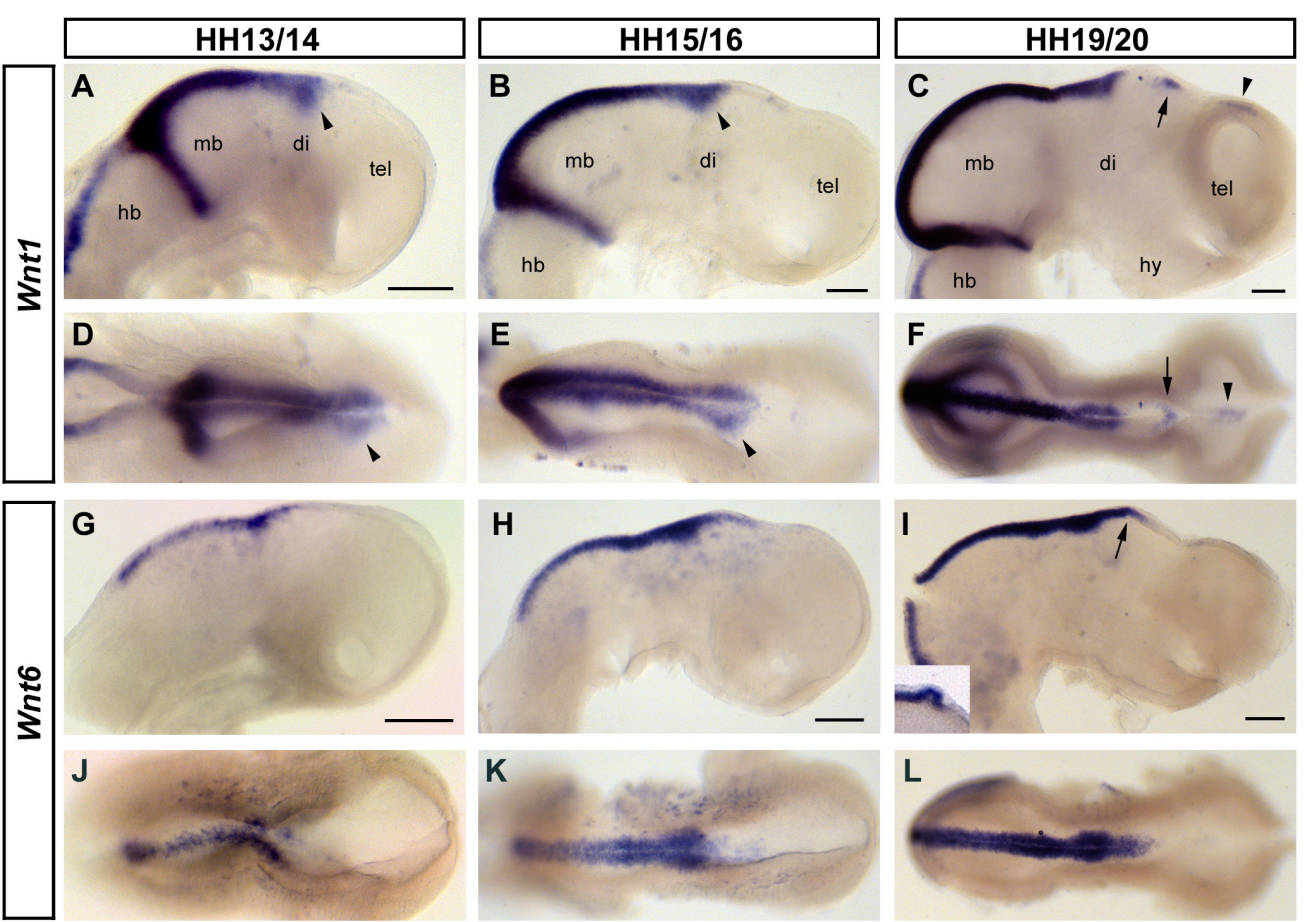
**Dorsalmost expression - *Wnt1 *and *Wnt6***. **(A-G) **Expression of *Wnt1 *(A-F) and *Wnt6 *(G-L) in HH13 (G,J), HH14 (A,D), HH15 (H,K), HH16 (B,E), HH19 (I,L) and HH20 (C,F) chick embryo brains. Lateral (A-C,G-I) and dorsal (D-F,J-L) views are shown; anterior points to the right. Note the broadening of *Wnt1 *expression at its anterior limit between HH14 and HH16 (arrowheads, A,B,D,E). Note expression of *Wnt1 *anterior to the epiphysis (arrow) and along the dorsal border of the pallium (arrowhead) at HH19 (C,F). Note the expression of *Wnt6 *in the evaginating pineal gland at HH19 (arrow and inset, I). Punctate expression of *Wnt6 *in (G-K) is likely to be in cranial ectoderm that was not completely removed from the neural tube [63]. Scale bars represent 0.2 mm. Abbreviations: di, diencephalon; hb, hindbrain; hy, hypothalamus; mb, midbrain; tel, telencephalon.

*Wnt1 *has previously been described at HH5 in a crescent-shaped domain in the anterior neural plate, suggesting a role in the induction or initial regionalisation of the forebrain [51], as well as at later stages where it is expressed in a stripe just anterior to the boundary between the midbrain and the hindbrain (MHB) and in two parallel lines flanking the roof plate of the midbrain and posterior diencephalon [62] (Figure 1A-F). Between HH14 and HH16, the expression that extends along the dorsal midbrain and into the posterior diencephalon laterally broadens at its anterior limit, which lies posterior to the prospective epiphysis (arrowheads, Figure 1A, B, D, E). This broadening is less apparent at later stages (Figure 1C, F). The area around the emerging epiphysis is free from *Wnt1 *transcripts; however, we have observed two previously undescribed regions of *Wnt1 *expression at HH20 - a distinctive patch in the epithalamic midline anterior to the epiphysis (arrow) and a region of weaker expression along the dorsal midline of the pallium (arrowhead, Figure 1C, F).

A previous study has described *Wnt6 *in the neural folds of the closing neural tube at the level of the prospective midbrain at HH7 and in the roof of the midbrain and posterior diencephalon at HH17 [63]. The anterior limit of neural crest production lies within the diencephalon [64]; thus, the expression pattern of *Wnt6 *is consistent with its suggested role as an inducer of the neural crest [65]. We found that *Wnt6 *is expressed in two narrow stripes flanking the *Wnt6*-negative roof plate in the dorsal midbrain and posterior diencephalon, highly reminiscent of *Wnt1 *expression in this area of the developing brain (Figure 1G-L). Unlike *Wnt1*, however, the anterior limit of *Wnt6 *expression coincides with the evaginating pineal gland (arrowhead and inset, Figure 1I).

### *Wnt3A*, *Wnt3 *and *Wnt4*

**Figure 2:**
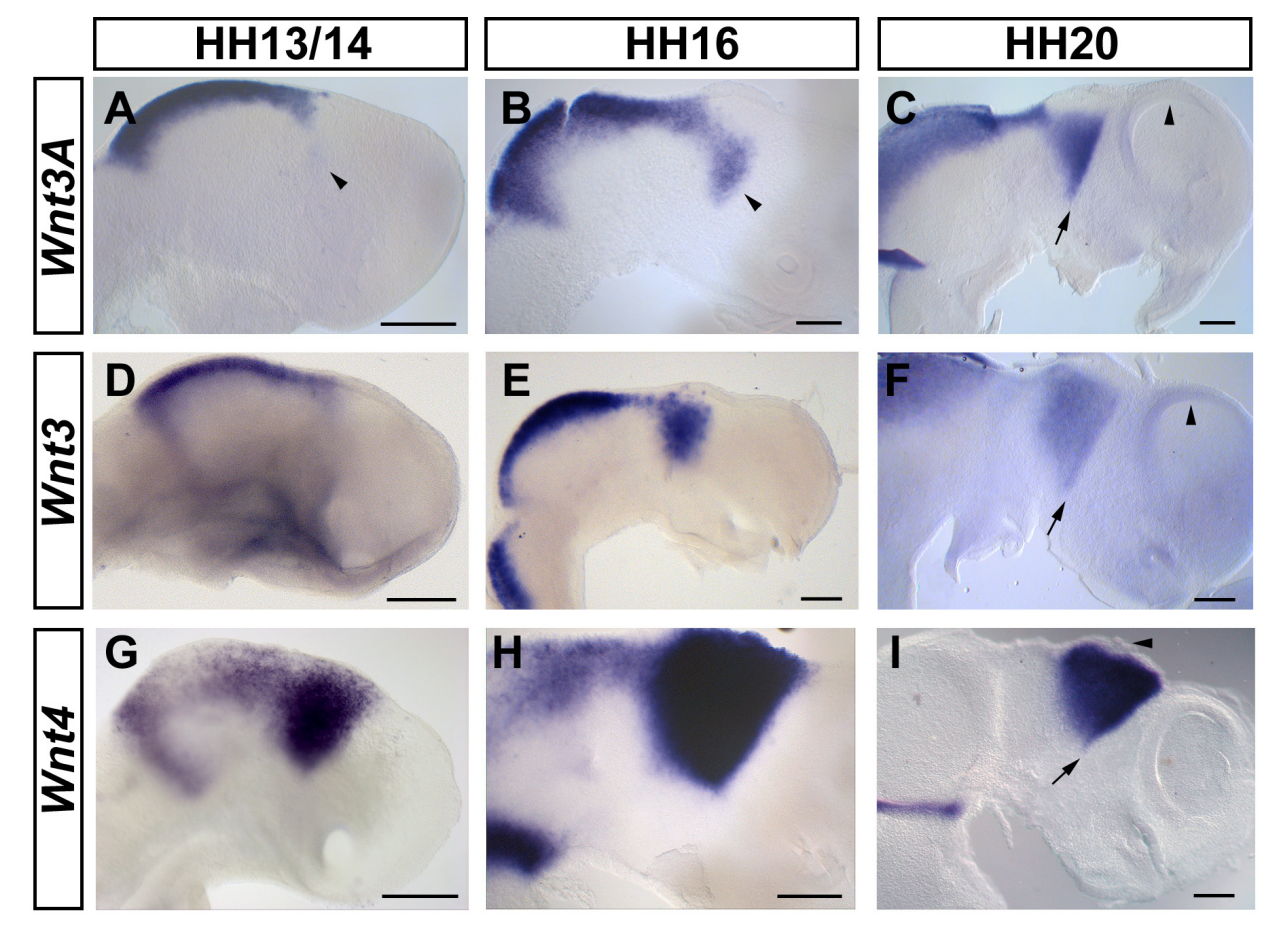
**Broader expression in the dorsal half of the forebrain - *Wnt3A*, *Wnt3 *and *Wnt4***. **(A-I) **Expression of *Wnt3A *(A-C), *Wnt3 *(D-F) and *Wnt4 *(G-I) at HH13 (A,D), HH14 (G), HH16 (B,E,H) and HH20 (C,F,I). Lateral views of whole mount (A,D,G) or hemisected brains (B,C,E,F,H,I) are shown; anterior points to the right. Note the expression of *Wnt3A *in the thalamus from HH13 onwards (arrowheads, A,B). Note the absence of clear *Wnt3/3A *expression from the dorsal pallium (arrowheads, C,F). Arrows mark the position of the zona limitans intrathalamica in (C,F,I). The arrowhead in (I) marks the epiphysis. Scale bars represent 0.2 mm.

According to a previous study, the first neuroepithelial expression of *Wnt3A *is found in the neural folds of HH8 embryos at the presumptive hindbrain level [66]. At later stages, both *Wnt3A *and *Wnt3 *are expressed along the dorsal midline of the neural tube with their anterior limit of expression in the posterior diencephalon (Figure 2A-F) [35,66].

The expression of *Wnt3A *and *Wnt3 *in the dorsal neural tube is similar to, but wider than that of, *Wnt1 *in this area. However, unlike *Wnt1*, both *Wnt3A *and *Wnt3 *are also expressed in the presumptive thalamus of the developing diencephalon: at HH13, a faint *Wnt3A *expression domain is detectable in this region (arrowhead, Figure 2A) that becomes increasingly strong until, by HH16, it begins to resemble the shape of an inverted triangle (arrowhead, Figure 2B). At this stage, *Wnt3 *is expressed in a very similar, if slightly broader and more diffuse, domain (Figure 2E). By HH20, the expression domains of both *Wnt3A *and *Wnt3 *in the presumptive thalamus have a sharply defined anterior limit at the ZLI (arrows, Figure 2C, F) and a less well-defined posterior limit. The area around the emerging epiphysis remains devoid of *Wnt3A *and *Wnt3 *transcripts at all stages examined. In contrast to the mouse embryo, where *Wnt3A *is found in the cortical hem [67], neither *Wnt3A *nor *Wnt3 *seem to be expressed along the dorsal border of the chick pallium (arrowheads, Figure 2C, F).

As shown previously, *Wnt4 *is broadly expressed in the dorsal half of the diencephalon and mesencephalon from HH7 onwards [66]. Subsequently, *Wnt4 *is downregulated in the prospective tectum and pretectum, but remains strongly expressed in a stripe just anterior to the MHB and within the developing thalamus and epithalamus (Figure 2G-I). Our study shows that, by HH20, *Wnt4 *is expressed in a distinctive wedge-shaped domain in the presumptive thalamus and epithalamus that is sharply delimited anteriorly at the ZLI (arrow, Figure 2I) and more diffusely defined posteriorly as described for *Wnt3A *and *Wnt3*. The emerging epiphysis appears to be free from *Wnt4 *transcripts (arrowhead, Figure 2I).

### *Wnt2B*, *Wnt5A*, *Wnt5B*, *Wnt7A*, *Wnt7B*, *Wnt8B *and *Wnt9A*

**Figure 3:**
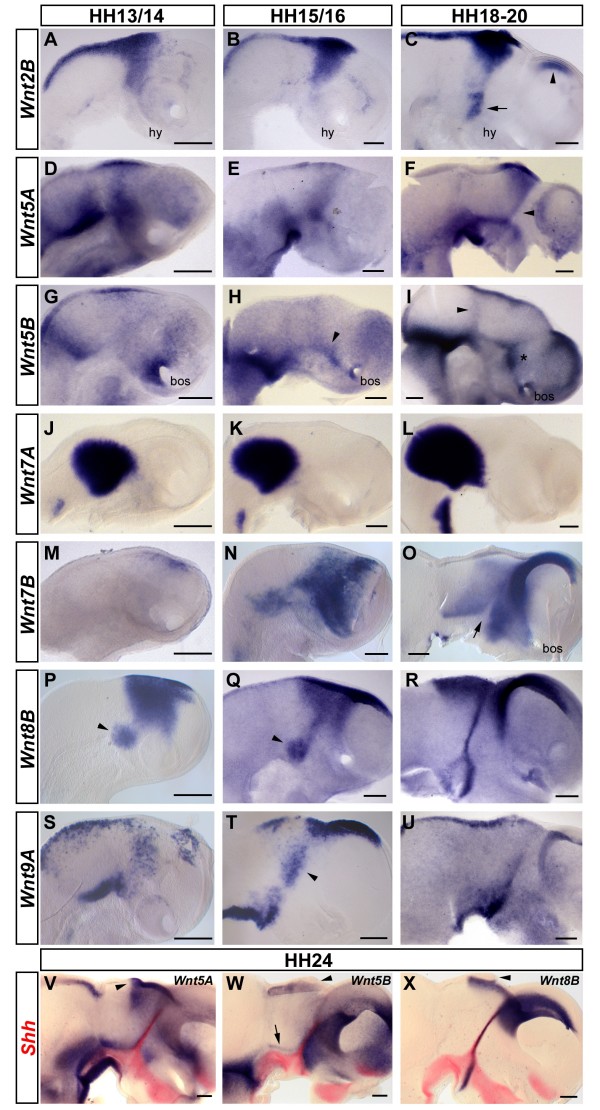
**Expression extending into the ventral forebrain - *Wnt2B*, *Wnt5A*, *Wnt5B*, *Wnt7A*, *Wnt7B*, *Wnt8B *and *Wnt9A***. **(A-X) **Expression of *Wnt2B *(A-C), *Wnt5A *(D-F,V), *Wnt5B *(G-I,W), *Wnt7A *(J-L), *Wnt7B *(M-O), *Wnt8B *(P-R,X) and *Wnt9A *(S-U) at HH13 (A,D,J,M,P), HH14 (G,S), HH15 (E,N), HH16 (B,H,K,Q,T), HH18 (I), HH19 (C,F,L), HH20 (O,R,U) and HH24 (double stainings with *Shh *in red, V-X). Lateral views of whole mount (A,D,J,M,P,S) or hemisected brains (B,C,E-I,K,L,N,O,Q,R,T-X) are shown; anterior points to the right. Note *Wnt2B *expression in the basal plate (arrow) and in the dorsal pallium at HH19 (arrowhead, C). The arrowhead in (F) highlights *Wnt5A *expression flanking the zona limitans intrathalamica (ZLI) at HH19. Note the expression of *Wnt5B *along the diencephalic dorsoventral interface at HH16 (arrowhead, H) and, at HH18, in the prethalamus (asterisk) and weakly at the DMB (arrowhead, I). The arrow in (O) marks the ZLI. Note the ventral patch of *Wnt8B *expression posterior to the hypothalamus between HH13 and HH16 (arrowheads, P,Q). Note the thalamic expression of *Wnt9A *lining up posterior to the prospective ZLI at HH16 (arrowhead, T). The arrowheads in (V-X) mark the evaginating pineal gland. Note the expression of *Wnt5B *in the thalamus at HH24 in a fine line parallel to the alar plate-basal plate interface (arrow, W). Scale bars represent 0.2 mm. Abbreviations: bos, base of optic stalk; hy, hypothalamus.

At all stages examined, *Wnt2B *is expressed along the dorsal midline of the midbrain and diencephalons, including the presumptive epiphysis (Figure 3A-C) [68]. In addition, *Wnt2B *expression expands into the prospective thalamus, forming an inverted triangle reminiscent of *Wnt3*, *Wnt3A *and *Wnt4*. Similar to *Wnt4*, this mid-diencephalic expression appears early and includes the epithalamus. By HH19, an additional domain of *Wnt2B *expression has appeared in the ventral diencephalon that is likely to mark the interface between the prospective Forel fields posteriorly and the hypothalamus anteriorly (arrow, Figure 3C). Furthermore, *Wnt2B *transcripts are now detected at the dorsal border of the pallium (arrowhead, Figure 3C).

Between HH13 and HH15, *Wnt5A *transcripts appear to be concentrated in the medial diencephalon, in the ventral midbrain (anterior stronger than posterior) and around the MHB (Figure 3D, E). At HH19, *Wnt5A *is strongly expressed in the epithalamus and this expression domain narrows ventrally to form a stripe flanking the ZLI (arrowhead, Figure 3F). A further stripe of expression is found along the alar/basal border between the posterior telencephalon and the midbrain. In the ventral midbrain, the expression of *Wnt5A *is more diffuse and appears to form a ventroanterior-to-dorsoposterior gradient (Figure 3F).

At HH14, *Wnt5B *transcripts are enriched at the MHB and around the base of the optic stalk, although weak expression is present throughout the diencephalon and in the ventroposterior pallium (Figure 3G). By HH16, *Wnt5B *has become upregulated along the border between the dorsal and ventral forebrain (arrowhead, Figure 3H) and weaker expression has spread throughout the pallium. By HH18, *Wnt5B *expression has been cleared from most of the dorsal midbrain while a strong area of expression remains present around the MHB (Figure 3I). Similar to *Wnt5A*, *Wnt5B *transcripts are found in the presumptive thalamus along both the ZLI and the alar/basal plate border. It is also expressed in the presumptive prethalamus (asterisk, Figure 3I) and in the dorsal telencephalon. Weak expression is detected around the diencephalon-midbrain boundary at this stage (DMB; arrowhead, Figure 3I) and the base of the optic stalk continues to express *Wnt5B*.

Between HH13 and HH19, *Wnt7A *is strongly expressed in a sharply contoured area that encompasses most of the midbrain and extends into the ventroposterior pretectum (Figure 3J-L). In contrast to the mouse embryo, where *Wnt7A *is widely expressed in the telencephalon [67,69], we could not detect any expression of *Wnt7A *in the anterior chick forebrain.

At HH13, *Wnt7B *is seen in a few cells in the dorsal forebrain (Figure 3M). By HH15, this expression has spread ventrally throughout the posterior telencephalon and most of the diencephalon. *Wnt7B *transcripts are also detected in the ventroposterior midbrain at this stage (Figure 3N). At HH20, *Wnt7B *is expressed in a rhomboidal domain coextensive with the presumptive thalamus similar to the expression of the homeobox gene *Gbx2 *in this area [54]. The midbrain, the basal plate ventral to the thalamus and the ZLI (arrow) are *Wnt7B*-free at this stage (Figure 3O). Anterior to the ZLI, *Wnt7B *is strongly expressed throughout the presumptive prethalamus and the eminentia thalami, in the dorsal telencephalon and in the posteriolateral hypothalamus [66,70]. This expression domain extends anteriorly up to the optic stalk and appears to include the posterior entopeduncular area (Figure 3O).

Previous studies have shown that *Wnt8B *is expressed in the neural plate during gastrulation [51] and that its interplay with the Wnt inhibitor Tlc regulates diencephalic versus telencephalic development in zebrafish [33]. We found that, by HH13, *Wnt8B *is expressed in a broad domain in the dorsal forebrain (Figure 3P). A distinctive patch of expression appears ventrally, posterior to the eye vesicle (arrowhead, Figure 3P). At HH15, *Wnt8B *transcripts are found in the presumptive epithalamus, the dorsal part of the thalamus and along the dorsoposterior border of the emerging pallium (Figure 3Q). The patch of expression in the ventral diencephalon is located posterior to the hypothalamus and is connected with the dorsal expression domain via a stripe of cells that is likely to correspond to the nascent ZLI (Figure 3Q) [70]. By HH20, this stripe has narrowed and the ventral expression domain has bifurcated into a stronger anterior and a weaker posterior stripe (Figure 3R).

At HH14, *Wnt9A *is expressed in a punctate fashion along the dorsal midline of the forebrain and midbrain, in the mid-diencephalic area and stronger in the ventral midbrain (Figure 3S). The mid-diencephalic expression becomes confined to a stripe of cells posterior to the forming ZLI after HH15 (arrowhead, Figure 3T, U). Expression in the dorsal telencephalon becomes stronger at this stage and marks the dorsal border of the pallium (Figure 3T, U).

In order to investigate their spatial relationship to the definitive ZLI, we performed double *in situ *hybridisation for *Wnt5A*, *Wnt5B *and *Wnt8B *with *Shh *on HH24 embryos. At this late stage, *Wnt5A *expression in the epithalamus and thalamus shows a sharply defined posterior limit at the level of the epiphysis (arrowhead, Figure 3V). Transcripts are also detected in the ventral prethalamus in an area that is likely to give rise to the zona incerta. In the telencephalon, there is diffuse expression throughout the pallium and in a spot at the base of the optic stalk. An AP gradient of *Wnt5A *expression is found in the roof of the midbrain with highest levels at the DMB. Comparison with *Shh *expression shows that the telencephalic and diencephalic basal plate (including the hypothalamus) are devoid of *Wnt5A *transcripts whereas *Wnt5A *overlaps with *Shh *expression in the ventral midbrain and that *Wnt5A *expression abuts that of *Shh *along the diencephalic alar/basal plate border and along the ZLI. Hence, areas of *Shh *expression in the forebrain are free from, but are flanked by, *Wnt5A *expression domains (Figure 3V).

At HH24, *Wnt5B *transcripts are absent from the diencephalic basal plate (Figure 3W). Similar to *Wnt5A*, the ZLI is flanked by areas of *Wnt5B *expression, but in contrast to *Wnt5A*, there is a small gap between the ventral stripe of *Wnt5B *expression in the thalamus and the *Shh*-expressing basal plate (arrow, Figure 3W). Whereas the entire thalamus expresses low levels of *Wnt5B*, the epiphysis is *Wnt5B*-free (arrowhead, Figure 3W). High levels of expression are observed in the prethalamus and along the dorsomedial telencephalon, extending far anteriorly into the lamina terminalis that is marked by *Shh*.

In contrast to *Wnt5A *and *Wnt5B*, *Wnt8B *is coexpressed with *Shh *in the ZLI (Figure 3X). *Wnt8B *expression has been cleared from most of the diencephalon at HH24 except for a dorsal domain in the epithalamus that surrounds the *Wnt8B*-negative epiphysis (arrowhead, Figure 3X). Strong *Wnt8B *expression is observed in the dorsomedial telencephalon at this stage while the posterior stripe of *Wnt8B *expression in the basal plate is no longer apparent.

### Wnt receptors - *Fz1*, *Fz2*, *Fz4*, *Fz7*, *Fz8*, *Fz9 *and *Fz10*

**Figure 4:**
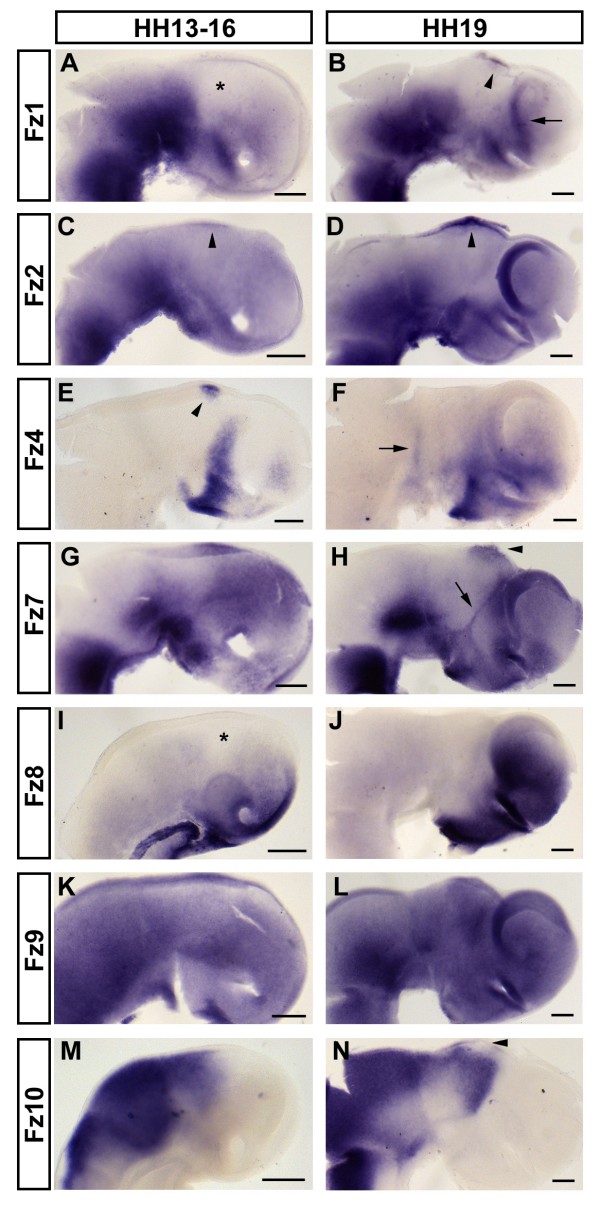
***Fz *expression**. (A-N) Expression of *Fz1 *(A,B), *Fz2 *(C,D), *Fz4 *(E,F), *Fz7 *(G,H), *Fz8 *(I,J), *Fz9 *(K,L) and *Fz10 *(M,N) at HH13 (I,M), HH14 (C,K), HH15 (A), HH16 (E,G) and HH19 (B,D,F,H,J,L,N). Lateral views of whole mount (M) or hemisected brains (A-L,N) are shown; anterior points to the right. Arrowheads mark epithalamic expression in (B-E,H) and absence of expression from the epiphysis in (N). Note the absence of *Fz *expression from the mid-diencephalic area (asterisks, A,I). Arrows mark the pallium-subpallium boundary, diencephalon-midbrain boundary and zona limitans intrathalamica in (B,F,H), respectively. Scale bars represent 0.2 mm.

Wnt signals are transduced via seven-pass transmembrane receptors of the Frizzled (Fz) family [71]. In a previous study, expression of *Fz1 *was found in the anterior neural plate of the gastrulating chick embryo [51]. At HH15, we detected strong expression of *Fz1 *in the ventral midbrain and ventroposterior diencephalon that fades out dorsally (Figure 4A). Weaker and diffuse expression is present in the telencephalon, whereas a large area in the anterior diencephalon - at the level of the emerging ZLI - appears to be free from *Fz1 *transcripts (asterisk, Figure 4A). Another domain of strong *Fz1 *expression is found ventroposterior to the base of the optic stalk at the interface between the presumptive subpallium and hypothalamus. The domains of strong *Fz1 *expression persist throughout HH19 (Figure 4B). In the telencephalon, *Fz1 *transcripts are concentrated in the posterior and ventral pallium and are enriched along the PSB by this stage (arrow, Figure 4B). Another expression domain has now appeared in the dorsal diencephalon with a posterior limit at the evaginating pineal gland (arrowhead, Figure 4B).

At HH14, *Fz2 *is strongly expressed in the ventral midbrain and diencephalon (Figure 4C). Whereas weaker and diffuse expression may be present throughout the forebrain, a distinctive domain of expression can be found in the dorsal diencephalon in the area of the prospective epiphysis (arrowhead, Figure 4C). By HH19, this expression domain has become more prominent (arrowhead, Figure 4D). In the ventral forebrain, *Fz2 *appears to be expressed at different levels in multiple stripes and patches by this stage, while an AP gradient of expression is detected within the emerging pallium (Figure 4D).

Expression of *Fz4 *has previously been described in the neural plate of the gastrulating chick embryo [51]. We have found that *Fz4 *is expressed in a surprisingly regionalised manner within the developing forebrain. By HH16, *Fz4 *transcripts form a distinctive band along the telencephalon-diencephalon interface that extends ventrally into the posterior hypothalamus (Figure 4E). It is also expressed in a small area in the epithalamus (arrowhead, Figure 4E) and in a weaker and more diffuse patch in the ventroanterior pallium. By HH19, *Fz4 *expression has spread within the anterior forebrain and is less sharply defined than at earlier stages (Figure 4F). The highest transcript levels are still detected in the posterior hypothalamus. An additional stripe of *Fz4 *expression has appeared along the DMB by this stage (arrow, Figure 4F).

At HH16, *Fz7 *is strongly expressed in three domains: in the ventral midbrain, ventral diencephalon and posterior hypothalamus. Weaker expression is detectable in most of the forebrain (Figure 4G). The ventral domains of strong *Fz7 *expression persist throughout HH19 (Figure 4H). At this stage, elevated levels of *Fz7 *are detected in the basal plate of the forebrain and in the ZLI (arrow, Figure 4H), precisely mirroring the expression of *Shh*. The epithalamus (including the evaginating pineal gland; arrowhead, Figure 4H), the eminentia thalami, the area posterior to the base of the optic stalk and the ventroanterior pallium also show higher levels of *Fz7 *expression.

By HH13, *Fz8 *transcripts are enriched in the ventroanterior forebrain, in the region of the presumptive hypothalamus and the lamina terminalis (Figure 4I). Weak expression may be present throughout most of the developing brain with the exception of a *Fz8*-free band in the centre of the diencephalon at the approximate level of the presumptive ZLI (asterisk, Figure 4I). By HH19, *Fz8 *expression is strong throughout the telencephalon (in a graded manner - high ventral to low dorsal - in the pallium) and in the ventroanterior hypothalamus.

Expression of *Fz9 *has been described before in neural precursors of the chick embryo [72]. We found this expression to be fairly ubiquitous at HH14 (Figure 4K), but by HH19, regions of increased *Fz9 *expression have appeared in the ventral midbrain and in the ventral pretectum as well as in a triangular region encompassing the epithalamus and the dorsal part of the thalamus (Figure 4L).

Previously, *Fz10 *has been described in the dorsal neural tube of the developing chick embryo [73]. By HH13, this dorsal expression has its anterior limit in the diencephalons, where it forms a triangular domain at the level of the presumptive thalamus. *Fz10 *transcripts are found throughout the midbrain (dorsal and ventral) at this stage (Figure 4M). By HH19, expression is found in the dorsal midbrain and diencephalon and in an inverted triangle in the presumptive thalamus, similar to the expression of *Wnt3*, *Wnt3A *and *Wnt4 *(Figure 4N). The epiphysis is free from *Fz10 *transcripts at this stage (arrowhead, Figure 4N). However, unlike *Wnt3 *and *Wnt3A*, *Fz10 *is also expressed in a DV stripe just anterior to the MHB and in the presumptive tegmentum. Weak expression of *Fz10 *is present throughout the midbrain, but not in the ventroposterior diencephalon, highlighting the DMB as a sharp interface between these two areas. Furthermore, a faint stripe of expression extends between the ventral tip of the thalamic triangle and the midbrain.

Taken together, it is clear that not only Wnt ligands, but also their receptors, are expressed in a highly regionalised and dynamic fashion in the developing forebrain.

### Extracellular and intracellular Wnt inhibitors - *Sfrp1*, *Sfrp2*, *Sfrp3*, *Axin1*, *Axin2 *and *Idax*

**Figure 5:**
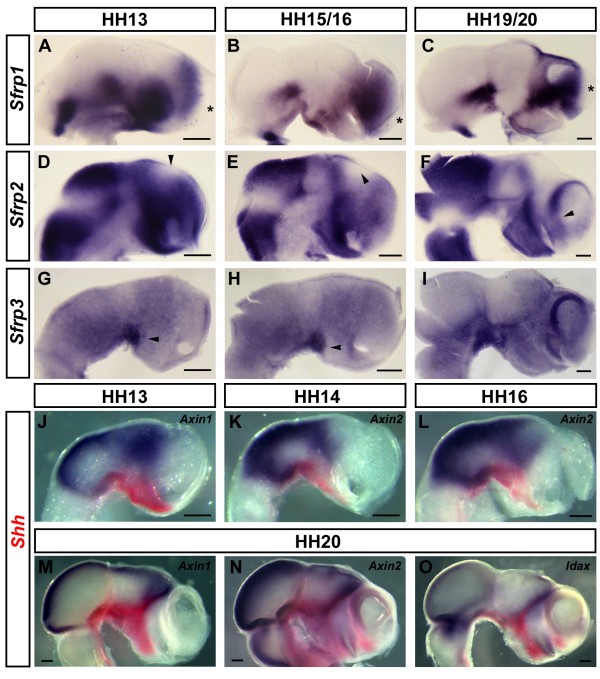
**Extracellular and intracellular Wnt inhibitors - *Sfrp1*, *Sfrp2*, *Sfrp3*, *Axin1*, *Axin2 *and *Idax***. **(A-O) **Expression of *Sfrp1 *(A-C), *Sfrp2 *(D-F), *Sfrp3 *(G-I), *Axin1 *(J,M), *Axin2 *(K,L,N) and *Idax *(O) at HH13 (A,D,G,J), HH14 (K), HH15 (H), HH16 (B,E,L), HH19 (C) and HH20 (F,I,M-O). (J-O) Double stainings with *Shh *in red. Lateral views of whole mount (A,D,J-O) or hemisected brains (B,C,E-I) are shown; anterior points to the right. Asterisks mark the commissural plate in (A-C). Note the absence of *Sfrp2 *expression from a dorsal patch between the telencephalon and diencephalon at HH13 and HH16 (arrowheads, D,E). The arrowhead in (F) indicates *Sfrp2 *expression in the pallium-subpallium boundary. Note the strong expression of *Sfrp3 *in the ventral diencephalon/midbrain (arrowheads, G,H). Scale bars represent 0.2 mm.

Wnt signals are modulated at multiple levels both extracellularly and intracellularly. Secreted Frizzled-related proteins (Sfrps) have a high sequence homology to the extracellular ligand-binding domain of the Frizzleds, the cystein-rich domain. They are thought to inhibit Wnt signalling by sequestering Wnt ligands in the extracellular space [74-76]. Previous studies have shown that both *Sfrp1 *and *Sfrp2 *are expressed in the neural plate during gastrulation, with *Sfrp2 *expression extending further posteriorly. Subsequently, *Sfrp1 *expression becomes restricted to the prospective forebrain whereas *Sfrp2 *is expressed broadly along the entire neuraxis [77-79]. At HH13, we found diffuse expression of *Sfrp1 *in the ventral midbrain and throughout the telencephalon with the exception of the prospective commissural plate (asterisk, Figure 5A). By HH16, expression in the ventral midbrain forms an anterior-to-posterior decreasing gradient (Figure 5B). At HH19, the posterior limit of *Sfrp1 *forebrain expression is more sharply defined and includes the anterior diencephalon (anterior epithalamus, eminentia thalami and anterior prethalamus). Ventrally, *Sfrp1 *is expressed at the posterior end and along the ventral midline of the hypothalamus, but it is absent from the anterolateral hypothalamus and from the area around the base of the optic stalk (Figure 5C). The commissural plate remains devoid of *Sfrp1 *transcripts at these later stages (asterisks, Figure 5B, C).

*Sfrp2 *is widely expressed in the developing brain. At HH13, transcripts are found throughout most of the forebrain and midbrain with the exception of the MHB and a dorsal region between the telencephalon and the diencephalon (arrowhead, Figure 5D). By HH16, domains of *Sfrp2 *expression are more clearly delineated (Figure 5E). By HH20, expression in the telencephalon has become confined to the dorsoposterior and lateral borders of the pallium, in particular the PSB (arrowhead, Figure 5F). In the diencephalon, *Sfrp2 *is expressed in the presumptive pretectum, in a diffuse diagonal stripe traversing the thalamus, in a sharply delineated line along the interface between dorsal and ventral forebrain and strongly in the prethalamus. Most of the epithalamus (including the epiphysis) and the eminentia thalami are free from *Sfrp2 *expression at this later stage. Strong expression of *Sfrp2 *is detected throughout the hypothalamus at all stages examined (Figure 5F).

Expression of *Sfrp3 *(also called *Frzb1*) has been described in the anterior neural plate of the gastrulation chick embryo [51]. At HH13, *Sfrp3 *is weakly expressed throughout the anterior neural tube (lower transcript levels in the presumptive pretectum), but a distinctive domain of strong expression is apparent in the ventralmost diencephalon (arrowhead, Figure 5G). This expression domain persists throughout HH15 (arrowhead, Figure 5H) and, by HH19, it is found at the border between the ventral diencephalon and midbrain (Figure 5I). *Sfrp3 *transcripts are enriched along the DMB at this stage and lower transcript levels are found throughout the forebrain. The presumptive pretectum remains the area with the lowest levels of *Sfrp3 *expression.

Axin is a cytoplasmic scaffolding protein that inhibits Wnt signalling by promoting glycogen synthase kinase 3β-mediated phosphorylation of β-catenin [80]. Between HH13 and HH20, both *Axin1 *and *Axin2 *are strongly expressed at the MHB and in a broad domain in the dorsal midbrain (Figure 5J-N). At HH13, *Axin1 *is expressed widely throughout the diencephalon whereas *Axin2 *is restricted to a narrower domain between the dorsoposterior and the ventral diencephalon (Figure 5J, K). By HH16, *Axin2 *is diffusely expressed throughout the diencephalon with a more defined anterior border at the level of the emerging ZLI (Figure 5L). Double *in situ *hybridisations with *Shh *show no significant *Axin *expression in the basal forebrain at the stages examined; however, their expression overlaps with *Shh *in the ZLI at HH19 (Figure 5M, N). *Axin1 *remains expressed at the MHB, along the dorsal midline between the MHB and the ZLI and in a wedge-shaped domain in the presumptive thalamus (Figure 5M). *Axin2 *is expressed in a similar, but broader, fashion with additional expression domains in the dorsal hindbrain, at the midbrain-forebrain boundary, along the dorsolateral roof of the pallium and around the base of the optic stalk (Figure 5N). The expression of *Axin1 *and *Axin2 *bears a striking resemblance to that of *Fz10 *in the forebrain and midbrain.

Idax is a cytoplasmic protein that antagonises Wnt signalling by preventing the interaction of Axin with the adaptor protein Dishevelled [81]. In *Xenopus laevis*, *Idax *is expressed in the anterior brain and is required for forebrain formation [17]. In the HH19 chick brain, we found *Idax *expression at the MHB, in a patch in the dorsoanterior diencephalon around the ZLI and in small domains anterior to the epiphysis in the dorsal epithalamus, around the optic stalk and weakly in the ventral pallium (Figure 5O).

### Transcriptional effectors and output of the Wnt pathway - *Lef1*, *Tcf1*, *Tcf3*, *Tcf4*, *Ctbp1*, *Ctbp2*, *Drapc *and *Axud1*

Members of the Tcf/Lef family of transcription factors (named after its founding members, T cell factor 1 and lymphoid enhancer factor) are known mediators of Wnt signals in the nucleus [71]. In the absence of a Wnt signal, Tcfs are thought to repress the transcription of target genes by recruiting co-repressors of the Groucho family. Activation of the Wnt pathway results in nuclear accumulation of β-catenin, which displaces Groucho and recruits the histone acetylase CBP/p300, thereby turning the Tcf complex into a transcriptional activator.

**Figure 6:**
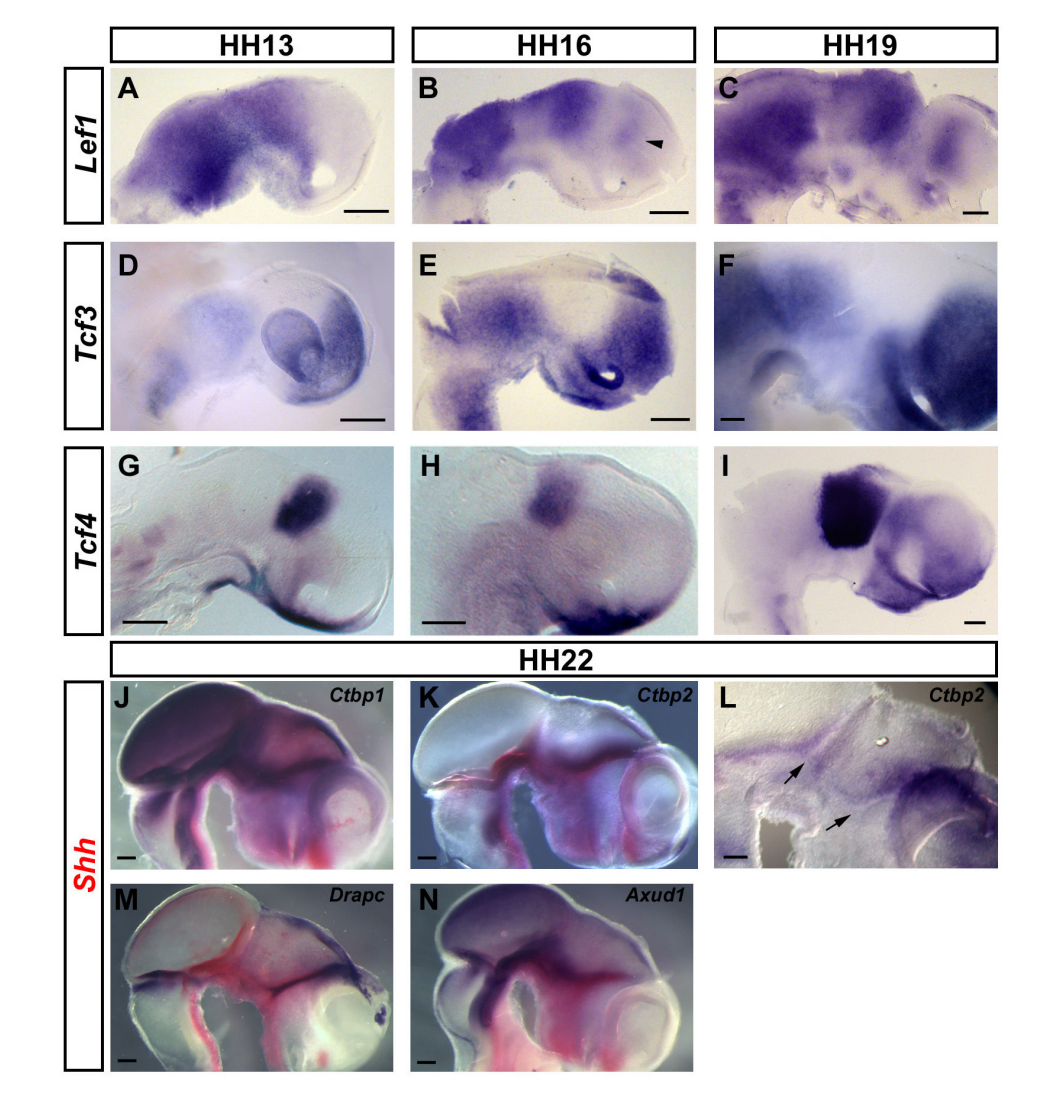
**Transcriptional effectors and output of the Wnt pathway - *Lef1*, *Tcf3*, *Tcf4*, *Ctbp1*, *Ctbp2*, *Drapc *and *Axud1***. **(A-N) **Expression of *Lef1 *(A-C), *Tcf3 *(D-F), *Tcf4 *(G-I), *Ctbp1 *(J), *Ctbp2 *(K,L), *Drapc *(M) and *Axud1 *(N) at HH13 (A,D,G), HH16 (B,E,H), HH19 (C,F,I) and HH22 (J-N). (J,K,M,N) Double stainings with *Shh *in red. Lateral views of whole mount (D,J,K,M,N) or hemisected brains (A-C,E-I,L) are shown; anterior points to the right. Note the *Lef1 *expression in the posterior pallium (arrowhead, B). Arrows mark the diencephalon-midbrain boundary and zona limitans intrathalamica in (L). Scale bars represent 0.2 mm.

Whereas *Tcf1 *is expressed ubiquitously in the developing brain between HH13 and HH19 (not shown), *Lef1 *transcripts are absent from the anterior forebrain at HH13 (Figure 6A). By HH16, domains of *Lef1 *expression begin to emerge more clearly (Figure 6B). High *Lef1 *levels are detected in the midbrain and presumptive thalamus whereas lower expression is found in the presumptive pretectum. An additional domain of *Lef1 *expression has started to appear in the prospective posterior pallium (arrowhead, Figure 6B). By HH19, expression in the posterior pallium has become stronger and seems to form an AP gradient (Figure 6C). Interestingly, *Lef1 *seems to become downregulated along regional interfaces within the neuroepithelium: the DMB, the interface between the diencephalon and the telencephalon as well as the interface between dorsal and ventral halves of the forebrain all have comparably low levels of *Lef1 *transcripts.

A previous study has shown that *Tcf3 *is strongly expressed in the anterior neural plate/tube from HH3 onwards [82]. At HH13, we detected transcripts in the telencephalon, midbrain and pretectum whereas a large part of the diencephalon - prospective epithalamus, most of the thalamus and part of the prethalamus - is free from *Tcf3 *expression (Figure 6D). Subsequently, the *Tcf3*-negative region in the centre of the forebrain appears to narrow down ventrally, giving rise to a wedge at the level of the presumptive ZLI that is complementary to the expression of *Wnt8B *in this area (Figure 6E, F). Interestingly, *Tcf3 *is also downregulated at the MHB.

*Tcf4 *is expressed in a distinctive stripe in the dorsal half of the posterior diencephalon as well as in the ventral telencephalon and hypothalamus at HH13 and HH15 (Figure 6G, H). The domain in the ventral forebrain encompasses the ventromedial hypothalamus and the preoptic area and may reach the lamina terminalis anteriorly. By HH19, *Tcf4 *is strongly expressed throughout the thalamus and the pretectum and more weakly in the prethalamus, hypothalamus, ventroposterior pallium and subpallium. The ventral diencephalon, the ZLI, the anterior epithalamus and the dorsal pallium are devoid of *Tcf4 *transcripts (Figure 6I).

C-terminal binding proteins (Ctbps) are transcriptional co-repressors that interact with Tcfs and mediate their repressive function, possibly by competing with β-catenin [83,84], although they may also act as transcriptional activators in certain contexts [85]. Expression of *Ctbp1 *and *Ctbp2 *in the chick neural plate/early neural tube has been reported elsewhere [86]. In order to analyse the expression of *Ctbps *in the forebrain at later developmental stages, we performed double *in situ *hybridisation with *Shh*. *Ctpb1 *is widely expressed in the HH20 chick brain with high levels of expression between the midbrain and the ZLI and low levels in the ventral telencephalon (Figure 6J). At HH20, weak *Ctbp2 *expression is detected at the MHB, along the alar/basal interface of the midbrain and diencephalon and at the DMB. Strong expression is found in a domain just posterior to the ZLI that fades towards the posterior and into the epithalamus. Lower transcript levels are detectable in the posterior prethalamus and in the pallium (Figure 6K, L). Taken together, it is interesting that *Ctbp2 *expression predominates in the vicinity of neuroepithelial boundaries (arrows, Figure 6L).

*Down-regulated by adenomatosis polyposis coli 1 *(*Drapc1*) is the orthologue of human *Adenomatosis polyposis coli down-regulated 1 *(*APCDD1*) and has been shown to be a target of Wnt signalling and a tumorigenic factor in colorectal carcinogenesis [87]. *Drapc *genes lack significant homology to other known gene families or conserved domains, but hydrophobicity algorithms suggest that they form double-pass transmembrane proteins [88]. In mice, *Drapc1 *is expressed in the prospective forebrain and midbrain areas from around the five-somite stage onwards. Between embryonic day 9.5 and 10.5, *Drapc1 *transcripts are found in the midbrain and diencephalon with highest levels of expression in the dorsal diencephalon and towards the ZLI as well as the MHB [88]. Because *Drapc *expression is thought to reflect Wnt pathway activation, we were interested in investigating its expression pattern in the chick forebrain. At HH20, *Drapc1 *is strongly expressed in the ZLI, at the MHB and along the dorsal midline of the diencephalon and pallium (Figure 6M).

*Axin-upregulated 1 *(*Axud1*)/*Cystein-serin-rich nuclear protein 1 *(*Csrnp1*) is a putative tumor suppressor gene that is upregulated by exogenous *Axin1 *expression in a colon cancer cell line [89,90]. It is a member of a family of three genes in mammals that are likely to act as transcriptional activators [90,91]. We were interested in examining the expression pattern of *Axud1 *in the developing forebrain, as it may represent another readout of Wnt pathway activity. At HH20, chick *Axud1 *is expressed in the midbrain and in the diencephalon up to the ZLI as well as around the base of the optic stalk, bearing resemblance to *Axin2 *expression (Figure 6N). Thus, it is likely that *Axud1 *is also a target of the Wnt/Axin signalling cascade during neural development.

### Multiple nested expression domains in the developing diencephalon

**Figure 7:**
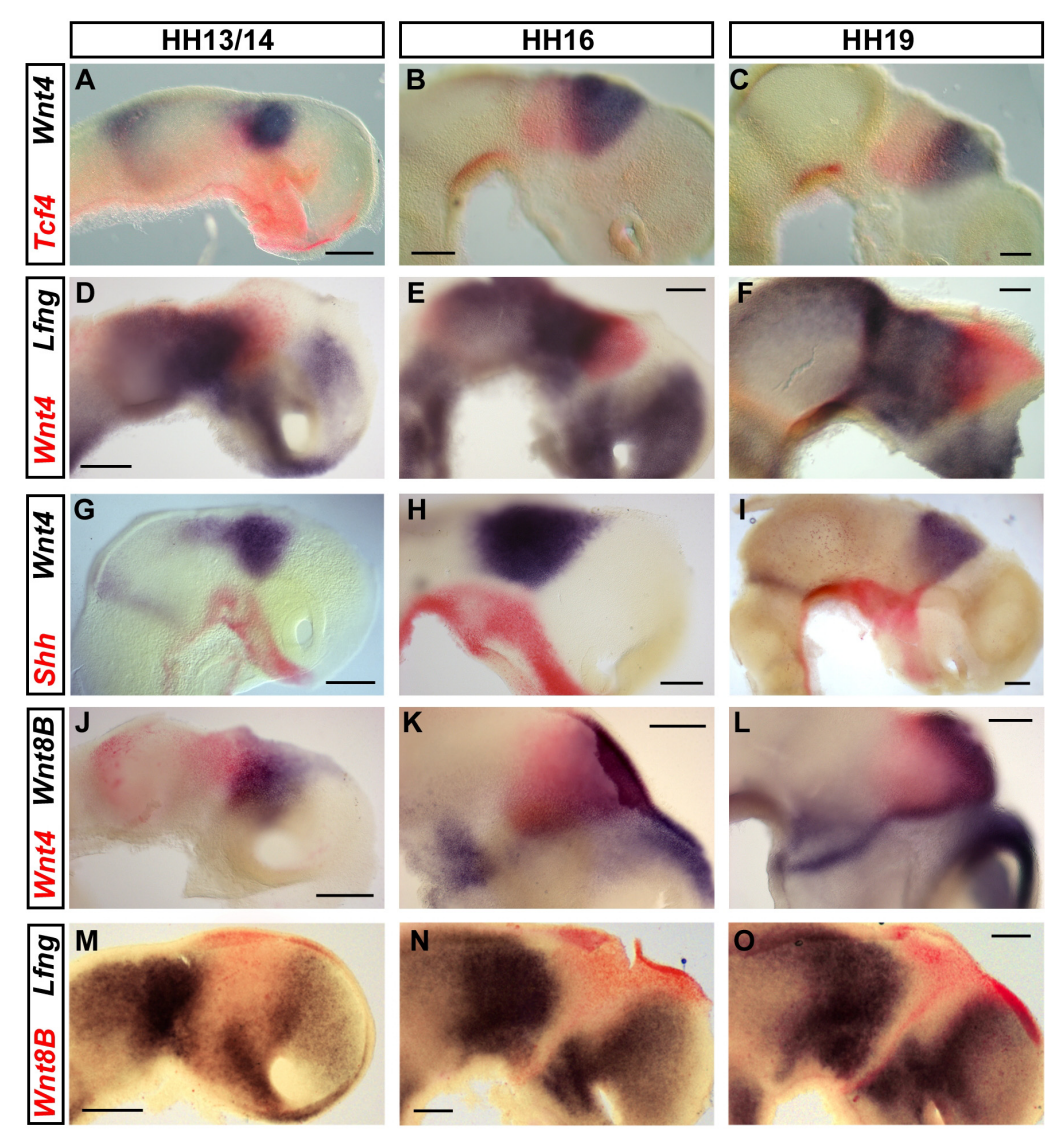
**Nested expression domains in the developing diencephalon**. **(A-O) **Double staining for *Tcf4 *(red) and *Wnt4 *(blue, A-C), *Lfng *(blue) and *Wnt4 *(red, D-F), *Shh *(red) and *Wnt4 *(blue, G-I), *Wnt4 *(red) and *Wnt8B *(blue, J-L), *Wnt8B *(red) and *Lfng *(blue, M-O) at HH13 (A,D,J,M), HH14 (G), HH16 (B,E,H,K,N) and HH19 (C,F,I,L,O). Lateral views of hemisected brains; anterior points to the right. Scale bars represent 0.2 mm.

A significant number of the genes examined in this study are expressed in distinctively shaped expression domains in the diencephalon, such as the stripe of *Tcf4 *that overlaps with the wedge-shaped domain of *Wnt4 *in the diencephalon (Figure 7A-C). This raises the question as to how these expression domains relate to the *Lfng*-free wedge in the early diencephalon that has previously been described and that gives rise to the ZLI at later stages [30]. For instance, we were interested to know whether the borders of expression of all these genes coincide or if they are expressed in a nested fashion. To investigate this further, we performed double *in situ *hybridisation of *Wnt4 *with *Lfng *and show that *Wnt4 *expression is seen within the *Lfng*-free wedge between HH13 and HH19 (Figure 7D-F). However, there is a narrow line of cells anterior to the *Wnt4 *domain that is free from both *Lfng *and *Wnt4 *expression. Posteriorly, *Wnt4 *overlaps with *Lfng *in the presumptive thalamus. The *Shh*-expressing ZLI appears to project into the anteriormost part of the *Wnt4 *domain (Figure 7G-I), suggesting that *Shh *is not expressed in the corridor of cells that is negative for both *Lfng *and *Wnt4*.

In the early forebrain, *Wnt8B *is expressed in a broad domain that covers, and is slightly wider than, the *Lfng*-free wedge (Figure 3M) [20,70]. Subsequently, expression narrows down to form a thin line at the level of the ZLI (Figure 3N, O). Double *in situ *hybridisation has confirmed co-localisation of *Wnt8B *and *Shh *in the ZLI (Figure 3U). The expression of *Wnt4 *and *Wnt8B *overlaps partially at all stages examined, with *Wnt4 *being expressed more posteriorly (Figure 7J-L). The expression of *Wnt8B *in the *Lfng*-free wedge at earlier stages [20] and in the ZLI at later stages (Figure 3U) raises the question as to whether *Lfng *and *Wnt8B *expression are inversely correlated. At HH13, *Wnt8B *expression covers the entire *Lfng*-free area in the diencephalon and, in fact, *Wnt8B *appears to overlap with *Lfng *- at least at the anterior expression interface (Figure 7M). However, the *Wnt8B *domain seems to narrow more rapidly than the gap in *Lfng *expression (Figure 7N) such that, by HH19, a fine line of *Wnt8B *expression is visible within the *Lfng*-negative corridor (Figure 7O; also see Figure 3X). Expression continues to overlap in the dorsoposterior pallium. Thus, Wnt pathway genes are expressed in multiple nested domains in the diencephalon that do not necessarily respect the proposed compartment boundaries at the emerging ZLI [30].

### *Wnt4 *expression in the thalamus is regulated by Shh signalling from the ZLI

**Figure 8:**
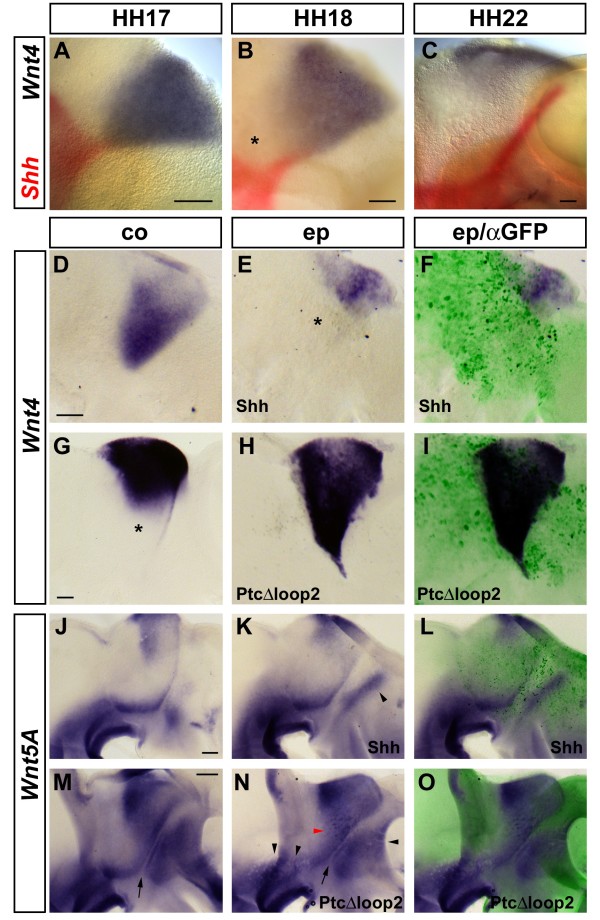
**Effects of Shh signalling on *Wnt4 *and *Wnt5A *expression in the developing forebrain**. Lateral views of hemisected brains; anterior points to the right. **(A-C) **Expression of *Shh *(red) and *Wnt4 *(blue) at HH17 (A), HH18 (B) and HH22 (C). Note the widening gap between *Wnt4 *expression and basal plate (B, asterisk). **(D-F) ***Wnt4 *expression in the control (co) half of the brain (D) and in the half that was electroporated (ep) at HH14 with Shh and green fluorescent protein (GFP)-expressing plasmids (E,F) after 24 hours of incubation. Note the downregulation of *Wnt4 *on the electroporated side (asterisk, E). (F) Overlay with anti-GFP staining. **(G-I) ***Wnt4 *expression in the control (co) half of the brain (G) and in the half that was electroporated (ep) at HH15 with PtcΔloop2 (H,I) after 48 hours of incubation. Note the ectopic expression of *Wnt4 *in (H) in an area of the thalamus that has downregulated *Wnt4 *on the control side (asterisk, G). (I) Overlay with anti-GFP staining. Scale bars in (A-D,G) represent 0.1 mm. (**J-L) ***Wnt5A *expression in the control (co) half of the brain (J) and in the half that was electroporated (ep) at HH14 with Shh and GFP-expressing plasmids (K,L) after 48 hours of incubation. Note the dorsal expansion of *Wnt5A *expression in the prethalamic area (arrowhead, K). **(M-O) ***Wnt5A *expression in the control (co) half of the brain (M) and in the half that was electroporated (ep) at HH15 with PtcΔloop2 (N,O) after 48 hours of incubation. Note the patches of *Wnt5A *downregulation in the ventral midbrain, ventroposterior diencephalon and posterior telencephalon (black arrowheads, N). Note the *Wnt5A *upregulation in a few cells in the thalamus (red arrowhead, N). Arrows mark the ZLI in (M,N). Scale bars in (J,M) represent 0.2 mm.

We have observed that the domain of *Wnt4 *expression in the presumptive thalamus retreats dorsally as *Shh *expression extends along the ZLI (Figure 7I). A closer examination of the expression of these two genes during the stages of ZLI formation by double *in situ *hybridisation reveals that the wedge of *Wnt4 *expression in the presumptive thalamus covers almost the entire DV extent of the diencephalic alar plate at HH17 (Figure 8A). However, a gap appears between the most ventral aspect of the *Wnt4 *expression domain and the basal plate at HH18, when *Shh *expression starts to project dorsally along the ZLI (asterisk, Figure 8B). By HH22, *Wnt4 *transcripts have been cleared entirely from the thalamic primordium and *Wnt4 *expression is only detectable dorsally in the epithalamus (Figure 8C). These observations prompt the question as to whether *Wnt4 *is negatively regulated by Shh signalling from the ZLI. To test if Shh has the potential to downregulate *Wnt4*, we electroporated a Shh-expressing plasmid into the diencephalon of pre-ZLI stage embryos (HH14 to HH16) *in ovo *[25]. Embryos were incubated overnight and analysed for the expression of *Wnt4*. In line with our hypothesis, 13 of 15 embryos showed a reduction of thalamic *Wnt4 *expression on the electroporated side (Figure 8D-F). To test whether Shh signalling is not only sufficient but also required to downregulate *Wnt4 *expression in the thalamus, we electroporated a mutant form of the Shh receptor Patched (Ptc^Δloop2^), which renders cells unresponsive to Shh signalling, into the diencephalon of HH14 to HH16 embryos [25], incubated them for 2 days and analysed the expression of *Wnt4*. Electroporated areas in the presumptive thalamus failed to downregulate *Wnt4 *expression compared to the contralateral side (n = 16; Figure 8G-I). These results indicate that Shh signalling from the ZLI is required to dowregulate *Wnt4 *in the presumptive thalamus.

Signals that are secreted from patterning centres in the developing brain often cross-regulate each other, mutually restricting their effect by forming negative feedback loops [92,93]. Using the *in ovo *electroporation approach, we tested whether Wnt signalling regulates *Shh *expression in the ZLI, but we found that the forced expression of various Wnt pathway activators or inhibitors had no influence on *Shh *expression in the diencephalon (data not shown).

### Differential regulation of *Wnt5A *expression by Shh signalling

We found that domains of *Shh *expression in the forebrain, in particular the ZLI and the diencephalic basal plate, are flanked by stripes of *Wnt5A *expression at later stages of brain development (Figure 3V). *Wnt5A *is known to be a Shh target in the murine hair follicle [94] and in basal cell carcinomas [95]; thus, we set out to test whether Shh signalling also regulates *Wnt5A *expression in the forebrain. *In ovo *electroporation of a Shh-expressing plasmid into the HH15 forebrain did not result in significant alterations of *Wnt5A *expression in the presumptive thalamus after 2 days of incubation (n = 18; Figure 8J-L). However, the expression domain of *Wnt5A *in the presumptive prethalamus is expanded dorsally and reaches into areas normally occupied by the eminentia thalami and the pallium in the majority of these embryos (14 of 18; arrowhead, Figure 8K). This expansion of the prethalamic *Wnt5A *expression domain does not strictly follow the pattern of the elctroporation (as indicated by the expression pattern of GFP). Hence, we assume that it reflects a general Shh-induced ventralisation of this brain area, rather than the direct induction of *Wnt5A *by Shh signalling.

Inhibiting Shh signal transduction by electroporating the blocking receptor PtcΔ^loop2 ^resulted in an even more complex outcome (Figure 8M-O): clusters of electroporated cells in the ventral midbrain and ventroposterior diencephalon as well as in the posterior telencephalon fail to express *Wnt5A*, indicating a requirement for Shh signalling in these areas (five of of nine embryos; black arrowheads, Figure 8N). In contrast, the stripes of *Wnt5A *expression that flank the ZLI (arrows, Figure 8M, N) appear unaffected by blocking Shh signal transduction (eight of nine embryos). Surprisingly, a few cells in the centre of the presumptive thalamus seem to upregulate *Wnt5A *expression in response to Shh inhibition (four of nine embryos; red arrowhead, Figure 8N). These observations suggest that the regulation of *Wnt5A *by Shh is regionally specific and may depend on other signals and/or regional competence factors.

Based on these gain- and loss-of-function experiments, we suggest that *Wnt5A *is unlikely to be a direct transcriptional target of Shh signalling in the thalamic primordium, unlike in the hair follicle or in basal cell carcinomas [94,95]. This notion is consistent with the presence of *Wnt5A *transcripts in the dorsal diencephalon before the onset of Shh secretion from the ZLI (Figure 3D, E).

## Discussion

Here, we have compiled an atlas of Wnt pathway gene expression in the chick forebrain between HH13 and HH24. We found that Wnt ligands fall into three groups with respect to their expression patterns in the forebrain: those that are expressed only in the most dorsal aspects of the forebrain (*Wnt1*, *Wnt6*), those that are more widely expressed in the dorsal half of the forebrain (*Wnt3*, *Wnt3A*, *Wnt4*) and those whose expression extends into the ventral forebrain (*Wnt2B*, *Wnt5A*, *Wnt5B*, *Wnt7A*, *Wnt7B*, *Wnt8B*, *Wnt9A*). Four *Wnt*s that we have examined are not expressed in the forebrain at the stages examined (*Wnt2*, *Wnt8*, *Wnt11*, *Wnt11B*). Areas that are particularly rich in *Wnt *expression are the dorsal midbrain and dorsoposterior forebrain, the thalamus and the dorsal border of the pallium (Table 1). This distribution is consistent with previous studies that have highlighted a requirement for Wnt signalling in the induction and expansion of dorsal neural identities [96-100], that have established that Wnts are required for thalamus formation [35,36], and that have postulated that the mammalian cortical hem is a signalling centre regulating the arealisation of the pallium [67].

**Table 1:** Regions of Wnt pathway gene expression in the embryonic chick forebrain

	**Pallium**	**PSB**	**Subpallium**	**Hypothalamus**	**Prethalamus**	**ZLI**	**Epithalamus**	**Epiphysis**	**Thalamus**	**Pretectum**	**Basal diencephalon**	**DMB**
*Wnt1*	HH19: dorsal	-	-	-	-	-	Dorsal	-	-	Dorsal only	-	-
*Wnt2B*	HH19: dorsal	-	-	Posterior end	-	+	++	++	Wedge	Dorsal only	Anterior end	-
*Wnt3*	-	-	-	-	-	+	Posterior	-	Wedge	Dorsal only	-	-
*Wnt3A*	-	-	-	-	-	+	Posterior	-	Wedge	Dorsal only	-	-
*Wnt4*	-	-	-	-	-	+	++	-	Broad wedge	Early stages	-	-
*Wnt5A*	Diffuse	+/-	Anterior	+/-	+	Flanking	+	+	+	Ventral only	HH17: vZLI	-
*Wnt5B*	+	+	Around bos	-	++	Flanking	Weak	-	Mostly weak	Weak	-	Weak
*Wnt6*	-	-	-	-	-	-	Dorsal	++	-	Dorsal only	-	-
*Wnt7A*	-	-	-	-	-	-	-	-	-	Ventropost.	Posterior	Ventral
*Wnt7B*	Dorsal	Posterior	Posterior	Posteriolat.	++	Flanking	+	-	+	Ventral	+/-	-
*Wnt8B*	Dorsal	-	-	Posterior end	-	++	+	-	-	-	Anterior	-
*Wnt9A*	Dorsal	-	-	-	-	+	Punctate	+/-	Punctate	Dorsal only	+	-
*Fz1*	AP gradient	++	+	++	+	-	Dorsoanterior	++	Ventropost.	Ventral	Posterior	Downreg.
*Fz2*	AP gradient	+	+	Posterior	Partly	Weak?	Dorsal	++	Ventropost.	Ventral	Patches	Downreg.
*Fz4*	+	+	+	Posterior	++	+/-	Spot	+/-	Weak	-	Weak	+
*Fz7*	+	+/-	+	++	Partly	++	+	+	Weak	Ventral	++	Downreg.
*Fz8*	++	++	++	Ventroant.	-	-	-	-	Weak?	Weak?	-	-
*Fz9*	+	+	+	+	+	Flanking	++	+	Wedge	Ventral	+	Downreg.
*Fz10*	-	-	-	-	-	Flanking	+	-	Wedge	Dorsal	-	+/-
*Sfrp1*	++	+	Posterior	Posterior	Anterior	-	Anterior	-	-	Ventropost.	Posterior	Ventral only
*Sfrp2*	Along border	++	Diffuse	++	++	Flanking	Weak post.	-	+	++	+	++
*Sfrp3*	+	++	+	+/-	+	+	Weak	-	Wedge	Weak	++	++
*Axin1*	-	-	-	-	-	+	+	-	+	Dorsal only	-	-
*Axin2*	HH19: weak	HH19: weak	HH19: weak	HH19: weak	HH19: weak	+	+	-	+	Dorsal	HH19: weak	+
*Idax*	-	Weak, diffuse	Around bos	+/-	+	+	Dorsoanterior	-	Anterior only	-	+/-	Weak
*Lef1*	AP gradient	+/-	+/-	Late	+	+	+	?	+	+	+/-	Weak
*Tcf1*	+	+	+	+	+	+	+	+	+	+	+	+
*Tcf3*	+ Excl. dors.	++	++	Anterior	+	-	-	-	Ventropost.	+	-	+
*Tcf4*	HH19: weak	Diffuse	Anterior	++	HH19: +	Flanking	+	+/-	++	++	-	-
*Ctbp1*	Weak	+/-	Weak	Weak	Weak	++	Dorsal	-	Stronger ant.	++	+	++
*Ctbp2*	+	+/-	+/-	+/-	Weak	Flanking	+	+/-	AP gradient	+	+/-	Flanking
*Drapc*	Dorsal	-	-	-	-	++	Dorsal	-	-	-	Weak	-
*Axud1*	+/-	+/-	+/-	+/-	+/-	+	Dorsal	-	Weak	Ventral	+/-	+

Abbreviations: ant., anterior; AP, anteroposterior; bos, base of optic stalk; DMB, diencephalon-midbrain boundary; dors., dorsal; downreg., downregulated; excl., excluding; HH, Hamburger and Hamilton; post., posterior; posteriolat., posteriolateral; PSB, pallium-subpallium boundary; ventroant., ventroanterior; ventropost., ventroposterior; vZLI, ventral extension of the ZLI in the basal plate; ZLI, zona limitans intrathalamica

### Canonical versus noncanonical Wnt signalling

Wnts can activate different intracellular pathways. Activation of the canonical Wnt pathway results in the stabilisation and accumulation of the multifunctional protein β-catenin and its subsequent translocation into the nucleus where it associates with factors of the Tcf/Lef family to activate the transcription of target genes [71]. During embryonic development, the canonical Wnt pathway regulates multiple cell fate decisions, from the induction of embryonic polarity before the onset of gastrulation [101,102] to organ specification [103]. Canonical Wnt signalling has also been implicated in oncogenesis [104] and stem cell maintenance [105].

Some Wnts are able to activate a noncanonical pathway that acts independently of transcription and regulates cellular polarity by affecting the actin cytoskeleton [106,107]. Because no transcriptional response is elicited, the output of the noncanonical pathway is more short-lived than that of canonical Wnt signalling. The noncanonical Wnt pathway is also referred to as the planar cell polarity (PCP) pathway because it plays a central role in regulating epithelial polarity - for example, in *Drosophila *where the orientation of sensory bristles is disturbed in PCP mutants and in the vertebrate inner ear where PCP signalling determines the orientation of stereociliar bundles (reviewed in [108]). Noncanonical Wnt signalling is also required for the convergent extension movements that mesodermal and neuroepithelial cells perform during gastrulation and that result in lengthening and narrowing of the body axis [109].

Although certain Wnts have been found to preferentially activate either the canonical or the noncanonical Wnt pathway, a comprehensive picture as to which Wnt induces which pathway in a specific cellular context remains elusive. While Wnt1, Wnt3A and Wnt8 are known activators of the canonical pathway in animal models and in cell culture experiments, Wnt5 and Wnt11 fail to elicit comparable effects in those tests [110-114]. However, both overexpression of *Wnt5*/*Wnt11 *or interference with their function in fish or frog embryos results in disturbed convergence and extension movements during gastrulation, establishing these two Wnts as *bona fide *activators of the PCP pathway [115-117]. Yet, both Wnt5 and Wnt11 have also been shown to activate the canonical pathway in the presence of certain receptors, suggesting that the specificity of pathway activation is regulated by the combination of ligands and receptors rather than simply by the type of Wnt molecule [118-121]. A recent study has demonstrated that Wnt5A/Wnt11 heterodimers activate the maternal β-catenin pathway that regulates axis formation in frog embryos, pointing towards a large number of possible ligand-ligand and ligand-receptor combinations that may activate either branch of the Wnt signalling pathway [122]. Furthermore, canonical and noncanonical Wnt signalling are known to antagonise each other, although it remains to be established whether this mutual inhibition has any biological significance (for example, [123]).

Of the *bona fide *activators of the noncanonical pathway, only *Wnt5A *and *Wnt5B *are expressed in the chick forebrain between HH13 and HH24, whereas neither *Wnt11 *nor *Wnt11B *are detectable at these stages. Interestingly, both *Wnt5A *and *Wnt5B *transcripts are enriched along regional interfaces and boundaries within the developing brain such as the MHB (*Wnt5B*), the ZLI (*Wnt5A *+ *Wnt5B*), the dorsal border of the pallium (*Wnt5B*) and the border between the alar plate and the basal plate (*Wnt5A *+ *Wnt5B*). It is tempting to speculate that noncanonical Wnt signalling may be involved in the morphogenesis of these boundaries that often form physical ridges or constrictions in the neuroepithelium. However, Wnt5A seems to function like Wnt1 in inducing dopaminergic neurons in the midbrain via the canonical Wnt pathway [124,125]. In addition to its well established role in epithelial morphogenesis, the PCP pathway has also been shown to regulate neuronal migration, axon guidance and dendrite morphogenesis [126], so Wnt5A and Wnt5B may also affect these processes at later stages of chick forebrain development.

The complexity of *Wnt *and *Fz *expression patterns during forebrain development highlights an urgent requirement to identify which pathway is activated in which brain area at a given developmental stage. Future studies could address this problem by analysing the activation pattern of intracellular factors that are exclusive to either the canonical or the noncanonical Wnt pathway.

### Inhibitors and transducers of Wnt signalling

*Sfrp1*, *Sfrp2 *and *Sfrp3 *are expressed in multiple domains in the developing brain, many of which overlap with the expression domains of various Wnt ligands. Thus, dynamic domains of Wnt inhibition may be superimposed on the already complicated pattern of Wnt activator expression. Usually, Sfrps are regarded as inhibitors of canonical Wnt signalling; however, we cannot rule out that they also interfere with the noncanonical Wnt pathway. Furthermore, anecdotal evidence has indicated that Sfrp1 functions in a biphasic manner, activating or inhibiting the Wnt pathway depending on dose [75]. Sfrps may act in a mutually antagonistic manner [127] and they bind not only to Wnts but also to Frizzleds [128,129], suggesting that their modulatory role in Wnt signalling is more complex than initially thought (reviewed in [130]). In addition, Sfrps interact with other Wnt-unrelated signalling pathways, most notably with the Bmp pathway where they function by inhibiting extracellular metalloproteinases of the Bmp1/Tolloid family that normally degrade the Bmp antagonist Chordin [131-134].

Recently, Sfrp1 has been shown to act as an axon guidance molecule by interacting with Fz2 independently of Wnt ligands [129]. Interestingly, we found a prominent patch of *Sfrp1 *expression at the ventral DMB - an area that is traversed by axons of the medial longitudinal fascicle [135]. Notably, several *Frizzleds*, including *Fz2*, are also expressed at high levels in this area (*Fz1*, *Fz2*, *Fz7*, *Fz9*). We speculate that Sfrp1 may be involved in guiding axons of this major longitudinal tract in the embryonic brain.

It is important to keep in mind that signalling receptors themselves can also limit the spread of a secreted signal and that they may be involved in removing a signal from the extracellular space [136]. Thus, the regionalised expression of multiple *Frizzleds *and *Sfrps *in the developing forebrain is likely to add an additional level of controlling Wnt ligand distribution.

In the mouse embryo, *Axin1 *expression has been reported to be ubiquitous [137] while *Axin2 *is known to be a target of Wnt signalling [138]. Hence, the expression of *Axin2 *is thought to be indicative of Wnt pathway activation. We found that the expression of *Axin1 *and *Axin2 *in the midbrain and posterior forebrain is very similar to that of *Wnt1*, *Wnt3 *and *Wnt3A *- *bona fide *activators of the canonical Wnt pathway - suggesting that canonical Wnt signalling is controlled by a negative feedback loop in this area of the brain. It is somewhat surprising that regions of the developing brain, such as the anterior forebrain and the hindbrain, which we show to express activators of the canonical Wnt pathway, do not express *Axin2*. On the one hand, it is well established that anterior neural development requires the repression of canonical Wnt signalling at early stages of development [11,16,139,140]. For example, the zebrafish *masterblind *phenotype that is characterised by an absence of the telencephalon and eyes is caused by a mutation in *axin1 *itself [12,13]. On the other hand, several Wnts that have been shown to activate the canonical pathway are expressed in the seemingly *Axin*-negative region of the anterior neural tube - at least at later stages (*Wnt7B*, *Wnt8B*, *Wnt9A*). More sensitive reporters may be useful to determine whether Wnt activation is really absent from *Axin*-negative areas of the developing brain.

Both *Lef1 *and *Tcf1 *are widely expressed throughout the developing forebrain, suggesting widespread competence to respond to Wnt pathway activation. The *tcf3 *gene is inactivated in the zebrafish mutant *headless *and it was shown that ectopic activation of Wnt targets, which would normally be repressed by Tcf3 at the anterior pole of the embryo, causes the microcephalic phenotype of this mutant [11]. The distinctive anterior domain of *Tcf3 *expression in the prospective telencephalon and anterior hypothalamus of the chick embryo is consistent with a requirement for Wnt repression in this area [16]. *Tcf3 *appears to be absent from the dorsal midline of the developing brain - a known Wnt signalling centre - and from the area encompassing the emerging ZLI, suggesting that this may also be a region of high Wnt activity. However, the Dishevelled antagonist *Idax *is expressed around the ZLI and may cap Wnt signalling intensity in this part of the diencephalon.

*Tcf4 *displays a surprisingly regionalised expression pattern in the developing forebrain. The expression domain in the ventral telencephalon and in the ventroanterior hypothalamus is consistent with a recent study in mouse where Tcf4 has been suggested to mediate the growth-promoting function of canonical Wnt signalling [141]. Another domain of strong expression is found in the posterior forebrain (thalamus + pretectum), which is known to depend on Wnt signalling [35,36]. In the spinal cord, Tcf4 determines the dorsal limit of the expression of the Shh target gene *Nkx2.2*, thereby integrating dorsal and ventral patterning signals [142]. It will be interesting to investigate to what extent Tcf4 antagonises Shh signalling from the ZLI. The graded expression of *Ctbp2*, encoding a Tcf-binding corepressor, posterior to the ZLI suggests a complex transcriptional response to Wnt signalling in the presumptive thalamus. The observation that various *bona fide *target genes of the canonical Wnt pathway (*Axin2*, *Axud1*, *Drapc*) are expressed in different patterns during brain development supports the idea that combinations of transcription factors and corepressors lead to divergent outputs of Wnt pathway activation.

### Expression domains of Wnt pathway genes in relation to forebrain subdivisions

Various models have been proposed for forebrain regionalisation in vertebrates. Based on the analysis of the expression of a large number of regional marker genes, it was suggested that the forebrain, similar to the hindbrain, develops in a segmented fashion. However, many of the proposed segmental interfaces do not fulfil the criteria for true compartment boundaries (reviewed in [20]). A study analysing the expression of various forebrain markers in HH8 to HH13 chick embryos failed to identify sharp boundaries or stable interfaces between expression domains at these early stages, indicating that regional marker genes are expressed dynamically and may not correlate with later segmental identities [143].

The most widely accepted model for forebrain development proposes that the diencephalon consists of three neural segments called prosomeres 1 to 3 (p1-3, from posterior to anterior) with p1 corresponding to the pretectum, p2 to the thalamus and p3 to the prethalamus [18]. Previous work in our lab has indicated that no cell lineage restriction is in force between the pretectum and the thalamus and between the prethalamus and the more anteriorly located telencephalon, discounting the idea of a complete segmentation of the diencephalon [54]. However, cell labelling studies have suggested that the ZLI develops from a relatively large, wedge-shaped area in the early forebrain anlage that is flanked by cell-tight boundaries both anteriorly and posteriorly and that is marked by the absence of *Lfng *expression [30]. Our double *in situ *hybridisation analysis using *Lfng*, *Shh*, *Tcf4*, *Wnt4 *and *Wnt8B *suggests that these regional markers are expressed dynamically in the diencephalon between HH13 and HH19 and that they do not in all cases respect regional boundaries. Most notably, *Wnt4 *expression reaches from the thalamus into the *Lfng*-negative triangle without respecting the posterior boundary of the prospective ZLI. Moreover, *Tcf4 *is expressed in a patch within the *Lfng*-free wedge; however, *Tcf4 *expression does not follow the apparent narrowing of this wedge and is still expressed in a broad stripe at HH19. Similarly, *Wnt7A *expression is found in a domain that does not respect the DMB. These observations suggest that diencephalic development is more dynamic than previously thought and highlight the interpretative pitfalls of equating gene expression domains with regional identities. However, it is remarkable that the expression of a large number of genes examined in this study at later stages is most sharply defined around the ZLI - a key boundary in the developing forebrain that has been suggested to mark the interface between prechordal and epichordal neuroepithelium [19,26,35].

### Differences between chick and mouse

In both chick and mouse, *Wnt1 *is expressed along most of the dorsal midline of the neural tube. However, while *Wnt1 *is excluded from the telencephalon and the anterior diencephalon in the mouse, we detected expression in the dorsal midline of these anteriormost brain regions at HH19. Interestingly, ectopic expression of *Wnt1*, similar to the expression described here in the anterior chick brain, is found in *Emx2 *mutant mice, indicating that the homeodomain transcription factor Emx2 normally serves to repress *Wnt1 *in the anterior brain of the mouse embryo. Such mice develop mild abnormalities in cortical layering that arise from defects in preplate development during early stages of cortical radial migration [144]. Since chick *Emx2 *is expressed in the anterior neural plate from HH8 onwards [143], the expression of *Wnt1 *in the anterior neural tube cannot be explained by the absence of this repressor. Thus, temporal differences in *Emx2 *expression between chick and mouse or a lack of Emx2 binding sites in the chick *Wnt1 *enhancer regions are likely explanations for the differences in *Wnt1 *expression.

The dorsal border of the pallium is a signalling centre that expresses multiple members of the Wnt family. However, while *Wnt3A *is strongly expressed in the cortical hem of the mouse embryo [67] and is required for the formation of the hippocampus [41], we could not find significant expression of *Wnt3A *or of its close homologue *Wnt3 *in the chick embryo (see also [35]). It is likely that other Wnts such as Wnt1 (not expressed in the murine cortical hem), Wnt2B, Wnt7B, Wnt8B and/or Wnt9A functionally replace Wnt3A in the dorsal telencephalon of the chick. Similarly, we detected only weak and diffuse expression of *Wnt5A *in the chick pallium after HH16, while mouse *Wnt5A *strongly marks the cortical hem [67].

*Wnt7A *expression has been reported in the mouse telencephalon, where it is required for the vascularisation of this brain region [69]. We found *Wnt7A *expression only in the chick midbrain and posterior diencephalon; hence, angiogenesis must be driven by another Wnt in the telencephalon of the chick embryo. As mentioned above, we also found a striking difference in the expression of an intracellular Wnt pathway antagonist: whereas mouse *Axin1 *is ubiquitously expressed during embryogenesis [137], we detected highly regionalised expression of the chick orthologue in the midbrain and posterior forebrain.

### Antagonism between Shh and Wnt signalling

*Shh *is expressed in the ventral midline along the length of the entire neural tube, consistent with its well characterised role in specifying different ventral neural identities in the spinal cord and brain [145,146]. Multiple *Wnts *are expressed along the dorsal midline of the neural tube, suggesting that they may exert a complementary role in specifying dorsal neural character [67,147-149]. It has been controversial whether Wnts truly act instructively by inducing dorsal neural identities [38-43,98,100,142] or whether they just promote proliferation of dorsal cell populations [43-48,97,99,150]. The treatment of neuroepithelial explants with Wnt proteins attenuates their transcriptional response to Shh, suggesting that these two classes of signalling factors act antagonistically in embryonic neuroepithelium [151]. Recently, it was shown that canonical Wnt signalling downregulates *Shh *expression in the ventral midbrain, thereby enabling neurogenesis in the otherwise non-neurogenic floor plate [152].

Two recent studies using chick *in ovo *electroporation have demonstrated that canonical Wnt signalling influences neural fates in the spinal cord by promoting transcription of the Shh antagonist *Gli3 *[153,154]. Conversely, Shh antagonises the dorsal expression of Wnts, as the Wnt-expressing cortical hem is lost in mice mutant for *Gli3 *[67]. An interaction of Shh and Wnt signalling has also been described in the somites [155,156], during tooth development [157] and in gastrula-stage *Xenopus *embryos [158], suggesting that these two pathways form a patterning module that is used reiteratively in different embryonic tissues. Moreover, the extracellular Wnt antagonists Sfrp1 and Sfrp2 are upregulated by Shh-Gli signalling in different experimental systems [159-161] and nuclear accumulation of the Wnt transducer β-catenin and the Shh transducer Gli1 are inversely correlated in a human colon cancer cell line [162]. Thus, the Shh and Wnt pathways antagonise each other involving both extracellular and intracellular mechanisms.

From HH18 onwards, *Shh *expression in the ZLI forms a distinctive peak protruding into the dorsal diencephalon and, for a long period until much later in development, this is the only non-ventral expression domain of *Shh *in the neural tube [30,163,164]. Given their predominantly antagonistic relationship, it is surprising that the expression domains of multiple *Wnts *are in close proximity to, or even overlap with, *Shh *expression in the ZLI. For example, both *Wnt3 *and *Wnt3A *are expressed in the thalamus and they are believed to be essential for thalamic specification [35]. The thalamic defects observed in *Lrp5*^-/- ^mice suggest that activation of the canonical Wnt pathway is required in this tissue [36]. *Shh *expression in the ZLI is also required for thalamic development [25-27], raising the question as to whether these two classes of signals act synergistically, rather than antagonistically, in the thalamic primordium. It is possible that the cellular competence to respond to combined Shh and Wnt signalling differs between the forebrain and the lateral spinal cord. Induction of the Shh target gene *Gbx2 *in the thalamus depends on the presence of Gli3, indicating a positive role of Gli3 in mediating Shh signalling in this area of the brain that is in contrast to its prevalent role as a Shh antagonist [165]. Since Glis are at the crossroads of the Shh and Wnt signalling pathways, this unusual role of Gli3 could suggest that Shh and Wnt signalling are integrated differently in the thalamus. Alternatively, it is conceivable that the two pathways do act antagonistically in the diencephalon as in other parts of the neural tube, but that they are required sequentially. Finally, it may simply be a tightly regulated balance between the two signals that results in proper thalamic specification.

Using *in ovo *electroporation, we found that Shh signalling from the ZLI is necessary and sufficient to downregulate *Wnt4 *expression in the presumptive thalamus, revealing an additional level of interaction between Shh and Wnts. We could not find any evidence for a converse regulation of *Shh *expression by canonical Wnt signalling (data not shown). Interestingly, *Wnt4 *has been identified as a target gene of Shh signalling in basal cell carcinomas whose mRNA levels are downregulated compared to normal skin [95]. Thus, the repression of *Wnt4 *by Shh may represent a conserved regulatory mechanism of clinical relevance.

Wnt4 has been implicated as an activator of the noncanonical Wnt pathway because its overexpression in zebrafish embryos results in gastrulation defects very similar to those produced by Wnt5, whereas none of the effects characteristic for activators of the canonical Wnt pathway are observed [166]. A reduction of *wnt4 *transcript levels using morpholino oligonucleotides enhanced the characteristic neurulation defects of *wnt5*/*wnt11 *mutant zebrafish embryos [167]. Wnt4 inhibits canonical Wnt signalling by redirecting β-catenin to the membrane, providing a mechanistic explanation for the frequently observed antagonism between the canonical and noncanonical Wnt pathways [168]. In combination with Frizzled3, Wnt4 has been implicated in axon guidance in the hindbrain, so it will be interesting to test whether *Wnt4 *exerts a similar role in the forebrain [169].

## Conclusion

Here, we have shown that a large number of genes associated with Wnt signalling are expressed in complex and highly dynamic domains during the development of the chick forebrain. Whereas Wnts are particularly abundant in certain areas - such as the dorsal midline of the neural tube and the thalamus - there are no areas in the forebrain that are completely devoid of *Wnt *expression, raising the question as to what extent and by what mechanism Wnts differ functionally. Furthermore, no areas were identified that exclusively express activators of either the canonical or the noncanonical pathway. Our study shows that the structural complexity of the vertebrate forebrain corresponds to the complexity of signalling events during its generation. In the future, it will be crucial to visualise the activation of canonical and noncanonical Wnt pathways *in vivo*, possibly by detecting the activation pattern of intracellular mediators of each pathway or by using particularly sensitive reporter assays.

We have analysed a number of genes with distinctive expression patterns in the diencephalon (*Lfng*, *Shh*, *Tcf4*, *Wnt4*, *Wnt8B*) by double *in situ *hybridisation and found that their expression boundaries often change their relative positions and do not necessarily respect proposed compartment boundaries and regional interfaces. This observation supports a dynamic model of diencephalic development in which regional identities cannot always be equated to gene expression domains and, more generally, it emphasises the necessity of complementing the molecular characterisation of neuroepithelial subdivisions with cell lineage analysis.

Diffusible signals that are involved in the regionalisation of the forebrain often interact. For example, Fgfs (expressed by the commissural plate), Shh (lamina terminalis), Bmps and Wnts (cortical hem) all cross-regulate each other during telencephalic arealisation [92,93,170]. We have identified a similar interaction in the diencephalon where Shh, secreted by the ZLI, negatively regulates *Wnt4 *expression in the prospective thalamus, resulting in a dorsalward retraction of the *Wnt4 *domain. A characterisation of the role of Wnt4 in forebrain development is now necessary to distinguish between direct effects of Shh on thalamic development and indirect effects that are mediated by the downregulation of *Wnt4*.

## Abbreviations

AP: anteroposterior; *Axud*: *Axin-upregulated*; Bmp: bone morphogenetic protein; Ctbp: C-terminal binding protein; DMB: diencephalon-midbrain boundary; *Drapc*: *Down-regulated by adenomatosis polyposis coli*; DV: dorsoventral; Fgf: fibroblast growth factor; Fz: Frizzled; GFP: green fluorescent protein; HH: Hamburger and Hamilton; *Lfng*: *Lunatic fringe*; MHB: midbrain-hindbrain boundary; PCP: planar cell polarity; PSB: pallium-subpallium boundary; Sfrp: secreted Frizzled-related protein; Shh: Sonic hedgehog; ZLI: zona limitans intrathalamica.

## Competing interests

The authors declare that they have no competing interests.

## Authors' contributions

RQ and CK designed the study, performed the gene expression analysis and drafted the manuscript. MG helped with *in situ *hybridisations. CK performed electroporation experiments and supervised the study. IM and AL provided funding for this research. All authors have approved the manuscript.
